# Surgical selection and outcomes: Unilateral hemilaminectomy vs. total laminectomy for spinal tumors

**DOI:** 10.1016/j.bas.2026.105944

**Published:** 2026-01-19

**Authors:** Almir Džurlić, Bekir Rovčanin, Džan Ahmed Jesenković, Azra Grebo, Lamija Terzić, Adi Ahmetspahić, Edin Hajdarpašić, Mirza Pojskić, Ibrahim Omerhodžić

**Affiliations:** a Department of Neurosurgery, Clinical Center University of Sarajevo, Sarajevo, Bosnia and Herzegovina; b Faculty of Medicine, School of Science and Technology, Sarajevo, Bosnia and Herzegovina; c Faculty of Medicine, University of Sarajevo, Sarajevo, Bosnia and Herzegovina; d Department of Neurosurgery, Philipps University Marburg, University Hospital Marburg, Germany

**Keywords:** Spinal tumors, Unilateral hemilaminectomy approach, Laminectomy, Postoperative outcomes

## Abstract

**Introduction:**

Spinal tumor surgery mandates complete removal with preserved neurological function and stability. Total Laminectomy (TL) provides access but risks complications (pain, deformity) from extensive tissue removal. The safer, tissue-sparing Unilateral Hemilaminectomy (UHL) is limited by concerns about complete resection via its narrower corridor.

**Research question:**

This study was comparing the clinical and radiological characteristic between unilateral TL and total laminectomy UHL and they clinical outcomes and complications.

**Material and methods:**

This was a retrospective cohort study comparing UHL and TL for intradural/extradural spinal tumors. We analyzed consecutive patients operated between January 2018 and December 2024, excluding those with confounding factors. Surgical approach was selected based on tumor location and intraoperative needs. Data on patient demographics, pre/postoperative neurological status, surgical parameters, and tumor characteristics were collected. Primary outcomes were postoperative neurological status and complications rate. Statistical analysis compared variables between groups using appropriate tests, with significance at p = 0.05.

**Results:**

Baseline characteristics were similar between groups, and the overall postoperative complication rate was low (6.3 %) and comparable. The postoperative KPS score between UHL and TL showed improvement, without significant difference between them. Both approaches yielded significant improvements in functional status and neurological recovery from preoperative baselines.

**Discussion and conclusion:**

Our findings indicate that the tissue-sparing UHL approach can achieve similar functional outcomes and complication rates as TL for similarly sized tumors. This supports UHL as a safe and effective option, although the final surgical approach must remain individualized based on specific tumor complexity and radiological findings.

## Highlights

• UHL and TL may have same results regarding clinical outcomes of patients.
• UHL and TL methods showed equally low rates of postoperative complications.
• Surgeons must individualize the approach based on the specific tumor.

## 1 Introduction

The surgical resection of spinal tumors is a complex endeavor that demands complete tumor removal while simultaneously preserving spinal stability and neurological function. Total laminectomy (TL) has historically served as the traditional and most common approach for achieving wide access to intradural and extradural lesions (Estefan et al., 2023) (Cattell and Clark, 1967). While effective for access, TL involves the bilateral removal of the lamina and posterior bony elements, necessitating the sacrifice of key stabilizing structures, including the supraspinous and interspinous ligaments and the paraspinal musculature (Cattell and Clark, 1967) (Chen et al., 2024a). This extensive soft and bony tissue destruction frequently results in severe postoperative complications, such as chronic axial pain and iatrogenic spinal deformity, which have been reported in up to 50 % of younger patients (Yasuoka et al., 1982; Liao et al., 2023). (Chen et al., 2024b; Estefan et al., 2023).

To mitigate these risks, unilateral hemilaminectomy (UHL) has emerged as a tissue-sparing, minimally invasive alternative, requiring the removal of only a unilateral portion of the lamina. UHL significantly reduces damage to bony and soft tissues, consequently offering a decreased risk of postoperative spinal instability (Mobbs et al., 2015; Pompili et al., 2016). (Fu et al., 2020),(Goodarzi et al., 2020a) Despite these protective benefits, the widespread adoption of UHL for spinal tumor resection has been limited. Surgeons have primarily restricted its use to small, laterally located tumors (Yasuoka et al., 1982) due to persistent concerns that the narrower surgical corridor may lead to incomplete tumor resection or increase the risk of spinal cord injury (Goodarzi et al., 2020; Yeo et al., 2011). (Goodarzi et al., 2020b).,(Lei et al., 2021)

Therefore, a critical need exists for comparative data to validate the efficacy and safety of the tissue-sparing UHL approach against the established TL method. The aim of our study is to investigate and compare the clinical and radiological characteristics and outcomes in patients treated with unilateral hemilaminectomy versus total laminectomy for intradural and extradural spinal tumors at our institution.

## 2 Material and methods

This was a retrospective cohort study conducted at our institution to compare unilateral hemilaminectomy and total laminectomy for the treatment of intradural/extradural spinal tumors. This study was conducted in accordance with the ethical principles of the Declaration of Helsinki. Since this was a retrospective study based on anonymized data, a waiver of informed consent was granted by the institutional ethics committee. Patient data confidentiality was strictly maintained throughout the research process.

The study population included all consecutive patients who underwent one of these two surgical approaches for intradural/extradural tumors between January 2018 and December 2024, older than 18 years. Exclusion criteria included patients with a history of prior spinal surgery, radiotherapy, or known spine metastatic disease, patients where biopsy was performed, as well as those with incomplete clinical data or who were not available for follow-up. All patients had undergone a preoperative spinal MRI with contrast. Unilateral hemilaminectomy or total laminectomy was performed, with the final selection influenced by the tumor's location and other intraoperative surgical requirements, according to the surgeon's preference.

Data were collected from patient medical records, including demographic details (age, sex), pre- and postoperative status at last follow-up (McCormick score, Karnofsky Performance Status (KPS)), and surgical parameters (operative time, hospitalization stay), tumor type, radiological tumor size and percentage of tumors occupying the intracanicular spinal space. Tumor size was measured on T1-contrast-enhanced MRI scans as the largest diameter in three orthogonal planes multiplied, then divided by 2. The percentage of tumors occupying the intracanicular spinal space was calculated on magnetic resonance on axial sequence using the following formula: (maximum tumor area in cm^2^)/(intracanicular space area in the same section in cm^2^) × 100 (%). The pathological diagnosis of tumors following surgery was confirmed by Department of pathology at our Institution according to World Health Organisation (WHO) Central Nervous system (CNS) tumor classification 2016 until 2021, and after according to WHO CNS 2021 tumor classification. Postoperative outcomes, including complications, and follow-up time, were also recorded. These variables were compared between two study groups (unilateral hemilaminectomy or laminectomy group). The primary outcomes observed in this study were postoperative neurological status and complications.

### 2.1 Operative technique

Patients were positioned prone under general anesthesia. The surgical level was determined using the C-arm. A midline incision was made in the skin following the identification of the desired surgical level. Intraoperative neuromonitoring was conducted by a functional neurologist for both surgical approaches. Furthermore, the TL approach served as a predetermined rescue strategy if the UHL provided inadequate exposure.

#### 2.1.1 Unilateral hemilaminectomy

**Fig. 1:**
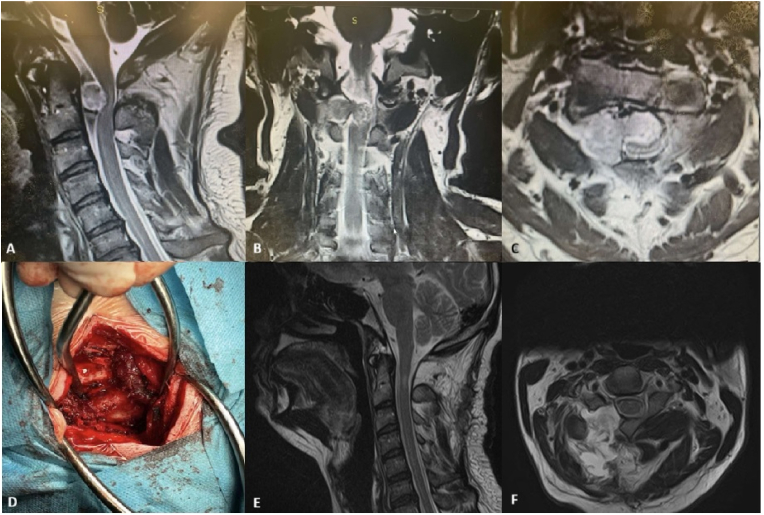
Resection of Dumbbell-Shaped Schwannoma via unilateral hemilaminectomy.

**Fig. 2:**
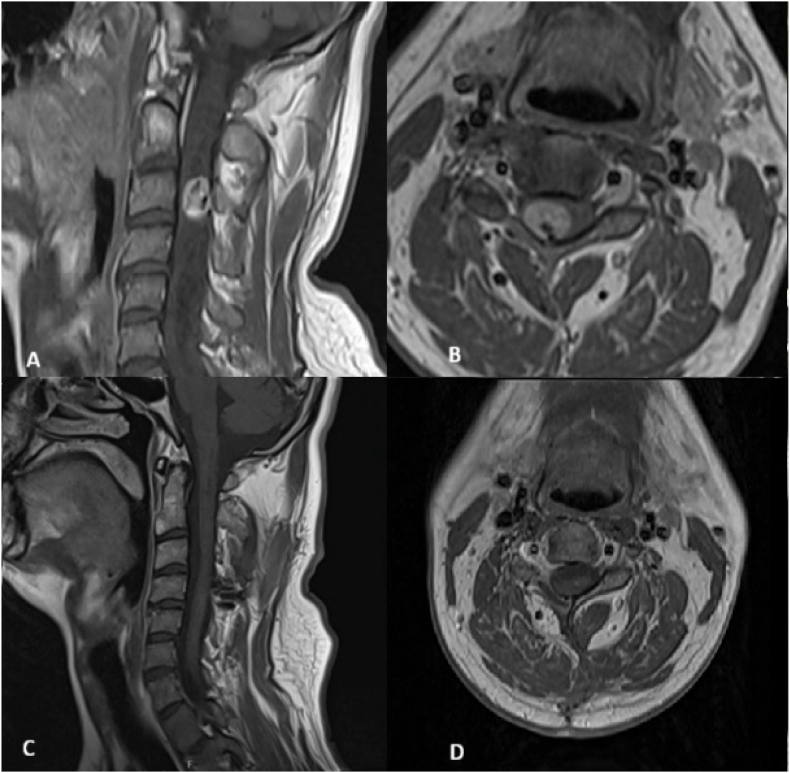
Resection of intramedullary hemangioblastoma via unilateral hemilaminectomy.

For the UHL, the muscle was dissected unilaterally along the subperiosteal plane to expose the lamina. By drilling the lamina, including the base of the spinous process, the dural sac was exposed while preserving the facet joint. We used several surgical techniques to overcome the limited view UHL. By undercutting the bases of the spinous processes and tilting the operating table obliquely to the opposite side, we were able to gain an adequate view for both the extradural and intradural procedures. For intradural lesions, a longitudinal paramedian incision was made in the dura, and the edges were tacked laterally to the muscle or fascia. The arachnoid membrane was sharply incised and then separated from the tumor. The tumor was removed through internal debulking of the solid mass or piecemeal resection, which also facilitated dissection. After the tumor was partially debulked, removing the remaining tumor mass from the spinal canal expedited the following steps. Tumors with cystic components were more easily addressed by draining the cystic fluid via puncture or aspiration. Dural and wound closer were performed in the standard way (Fig. 1, Fig. 2).

#### 2.1.2 Laminectomy

**Fig. 3:**
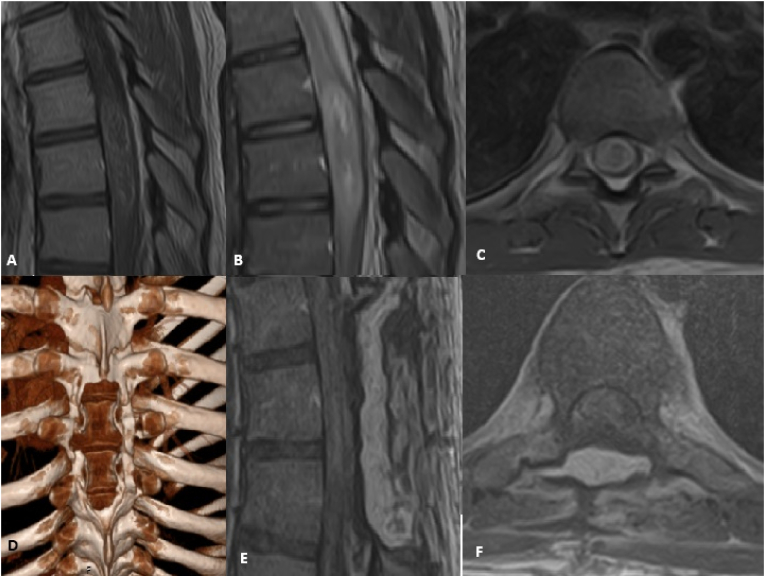
Diffuse Midline Glioma of the Thoracic Spinal Cord in a 20-Year-Old Patient Treated by Laminectomy approach. A: Preoperative sagittal T1-weighted MRI with contrast demonstrating the intramedullary lesion (DMG) with heterogeneous enhancement. B: Preoperative sagittal T2-weighted MRI showing the hyperintense intramedullary tumor with extensive spinal cord edema superiorly and inferiorly to the lesion. C: Preoperative axial T1-weighted MRI with contrast confirming the intramedullary location and enhancement pattern of the glioma. D: Postoperative 3D Computed Tomography (CT) reconstruction illustrating the surgical decompression: partial laminectomy of TH6 and total laminectomy of TH7 and TH8. E: Early postoperative sagittal T1-weighted MRI with contrast showing the resection cavity, changes related to the surgery, and the epidural placement of an autologous fat graft (arrowhead) used to prevent epidural fibrosis. F: Early postoperative axial T1-weighted MRI with contrast demonstrating the relationship of the resected area and the epidural fat graft (arrowhead) to the spinal cord.

**Fig. 4:**
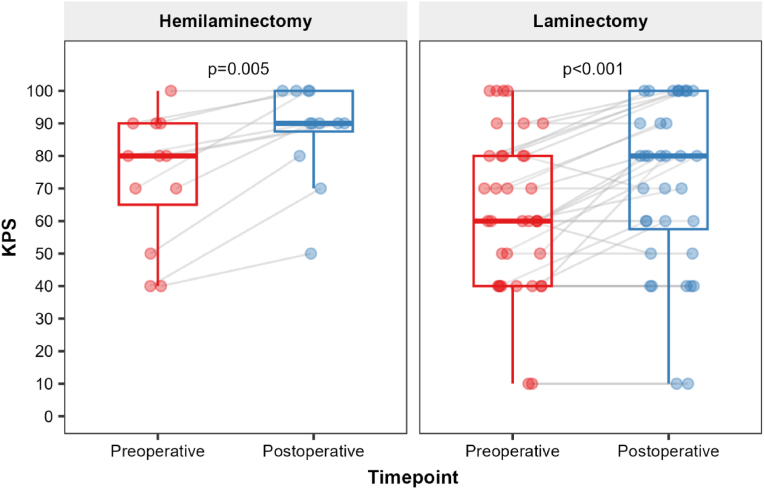
Box-plot diagram of KPS scores between timepoints across groups.

For laminectomy, paravertebral muscle dissection was bilateral, a high-speed pneumatic drill was used to resect the laminae, the supraspinal and interspinous ligaments were dissected, and the spinous process ligament complex was completely removed. Subsequently, the dura was opened, and micro-neurosurgical techniques were used to resect the tumors. To prevent blood clots from entering the subarachnoid space, cottonoids were placed at both the upper and lower poles. Subsequently, the dural sac was sealed with uninterrupted sutures. Standard closing of the wound was performed (Fig. 3) (see Fig. 4).

### 2.2 Statistical analysis

Statistical analysis was conducted in R 4.5.1 (R Foundation for Statistical Computing, Vienna, Austria). Descriptive summaries are reported as mean (±SD), median [Q1-Q3], and range (min-max) for numerical variables. Ordinal and categorical variables are reported in absolute and relative counts within each stratum, and ordinal variables are summarized as median [Q1-Q3]. Significance threshold was set at p = 0.05, with lower p-values deemed as statistically significant at this level. For quantitative variables, differences in means between groups were tested with independent samples Welch *t*-test or Wilcoxon rank sum test, as deemed appropriate by assumptions about the implied variables. Differences in paired samples were tested with a paired Wilcoxon signed rank test. Confidence intervals for the median differences were estimated using non-parametric bootstrap resampling with 10,000 resamples, and ordinal paired test was done using Marginal homogeneity test (Stuart-Maxwell test).

A 36-year-old male presenting with progressive spastic quadriparesis due to a C1-C2 dumbbell-shaped extradural and intradural extramedullary schwannoma. Preoperative diagnostic imaging shows the tumor (A: Sagittal T2-weighted MRI, B: Coronal T2-weighted MRI, C: Axial T2-weighted MRI). D: Intraoperative image illustrating the minimally invasive unilateral hemilaminectomy approach. Postoperative follow-up MRI (E: Sagittal T2-weighted MRI, F: Post-contrast Axial T1-weighted MRI) confirms total tumor excision and resolution of spinal cord edema, demonstrating the UHL technique's ability to achieve oncological clearance, maintain spinal stability, and facilitate rapid neurological recovery.

A 39-year-old male presented with progressive right-hand weakness due to an intradural intramedullary hemangioblastoma at the C3 level. Preoperative imaging reveals the lesion (A: Sagittal T2-weighted MRI, B: Axial T2-weighted MRI). The tumor was completely removed en-bloc using a minimally invasive unilateral hemilaminectomy (C3-C4) combined with the posterolateral sulcus technique. Postoperative follow-up MRI (C: Sagittal T2-weighted MRI, D: Axial T2-weighted MRI) confirms total tumor resection and highlights the preservation of posterior midline structures, leading to immediate improvement in motor strength and full symptomatic relief.

## 3 Results

The study included a total of 48 patients, consisting of 12 patients in the UHL group and 36 patients in the TL group. No surgical transformation from UHL to TL was observed. Descriptive statistics for all baseline variables are presented in Table 1. The two surgical cohorts were well-matched, as no statistically significant differences were observed for any baseline demographic or clinical characteristic evaluated. Specifically, patient age, with an overall mean of 50 ± 18 years, was comparable between groups (p = 0.13). Sex distribution was also balanced overall and between the groups (p = 0.5), with 50.0 % of the overall cohort being female and 50.0 % male. Furthermore, there were no significant differences in the primary location of the surgery (p > 0.9), with the thoracic spine being the most frequent site (58.3 %). Preoperative functional and neurological status, assessed by the preoperative KPS (p = 0.15) and preoperative McCormick score (p = 0.9), were also comparable across the UHL and TL groups.

**Table 1:** Patient characteristics.

	Surgery type			
	Overall N = 48^a^	Hemilaminectomy N = 12^a^	Laminectomy N = 36^a^	p-value
**Age**				0.13^b^
Mean (±SD)	50 (±18)	56 (±14)	48 (±19)	
Median [Q1-Q3]	52 [38–65]	61 [44–67]	48 [37–63]	
Min - Max	10–79	31–70	10–79	
**Sex**				0.5^c^
Female	24 (50.0 %)	7 (58.3 %)	17 (47.2 %)	
Male	24 (50.0 %)	5 (41.7 %)	19 (52.8 %)	
**Site of surgery**				>0.9^d^
Cervical	9 (18.8 %)	2 (16.7 %)	7 (19.4 %)	
Thoracic	28 (58.3 %)	7 (58.3 %)	21 (58.3 %)	
Lumbar	11 (22.9 %)	3 (25.0 %)	8 (22.2 %)	
**Localisation**				0.2^d^
Extradural	7 (14.6 %)	3 (25.0 %)	4 (11.1 %)	
Intradural extramedullary	28 (58.3 %)	5 (41.7 %)	23 (63.9 %)	
Intradural extramedullary + Extradural	3 (6.3 %)	2 (16.7 %)	1 (2.8 %)	
Intradural intramedullary	10 (20.8 %)	2 (16.7 %)	8 (22.2 %)	
**Preoperative McCormick score**				0.9^d^
1	5 (10.4 %)	1 (8.3 %)	4 (11.1 %)	
2	17 (35.4 %)	5 (41.7 %)	12 (33.3 %)	
3	11 (22.9 %)	3 (25.0 %)	8 (22.2 %)	
4	11 (22.9 %)	3 (25.0 %)	8 (22.2 %)	
5	4 (8.3 %)	0 (0.0 %)	4 (11.1 %)	
**Tumor volume (cm**^c^**)**	4.5 (±4.6)	5.7 (±6.6)	4.1 (±3.7)	0.4^b^
**Preoperative KPS**	70 [45–80]	80 [60–90]	60 [40–80]	0.2^e^
**Occupied intracanicular space (%)**	63.3 (±19.4)	60.5 (±18.1)	64.2 (±19.9)	0.6^b^
**Tumor type (structured)**				0.8^d^
Meningioma	13 (27.1 %)	4 (33.3 %)	9 (25.0 %)	
Schwannoma	12 (25.0 %)	4 (33.3 %)	8 (22.2 %)	
Other tumors	8 (16.7 %)	2 (16.7 %)	6 (16.7 %)	
Ependymoma	5 (10.4 %)	0 (0.0 %)	5 (13.9 %)	
Hemangioblastoma	4 (8.3 %)	1 (8.3 %)	3 (8.3 %)	
Glioma	3 (6.3 %)	0 (0.0 %)	3 (8.3 %)	
Lymphoma	3 (6.3 %)	1 (8.3 %)	2 (5.6 %)	

a n (%); Mean (±SD); Median [Q1-Q3].

b Welch Two Sample *t*-test.

c Pearson's Chi-squared test.

d Fisher's exact test.

e Wilcoxon rank sum test.

Surgical outcomes, summarized in Table 2, showed that both groups had a low rate of postoperative complications (6.3 % overall) with no significant difference between them. In Postoperative complications in the UHL group included one case of wound dehiscence on the 10th day following suture removal. In the TL group, one patient with an intramedullary glioblastoma developed syringomyelia and hydrocephalus requiring ventriculoperitoneal shunt placement; another patient experienced a pseudomeningocele (CSF collection) following tumor resection, which was managed conservatively.

**Table 2:** Surgical outcomes by surgery types.

	Surgery type			
	Overall N = 48^1^	Unilateral Hemilaminectomy N = 12^1^	Total Laminectomy N = 36^1^	p-value
**Postoperative complications**	3 (6.3 %)	1 (8.3 %)	2 (5.6 %)	>0.9^2^
**Postoperative McCormick score**				0.070^2^
1	16 (33.3 %)	4 (33.3 %)	12 (33.3 %)	
2	17 (35.4 %)	8 (66.7 %)	9 (25.0 %)	
3	7 (14.6 %)	0 (0.0 %)	7 (19.4 %)	
4	4 (8.3 %)	0 (0.0 %)	4 (11.1 %)	
5	4 (8.3 %)	0 (0.0 %)	4 (11.1 %)	
**Postoperative KPS**	80 [60–100]	90 [85–100]	80 [55–100]	0.081^3^
**Operative time (min)**	155 [120–200]	150 [120–185]	165 [120–210]	0.6^3^
**Hospitalization time (days)**	8 [6–13]	7 [6–10]	8 [6–14]	0.7^3^
**Postoperative follow-up time (months)**	7 [2–17]	14 [12–18]	6 [2–14]	**0.031^3^**

^1^n (%); Median [Q1-Q3].

^2^Fisher's exact test.

^4^Wilcoxon rank sum test.

Functional status improved after surgery in both groups (Table 3). In the UHL group (N = 12), the mean KPS increased from 73.3 (±20.2) preoperatively to 87.5 (±14.8) postoperatively; the median increased from 80 [60–90] to 90 [85–100], with values ranging from 40 to 100 preoperatively and 50–100 postoperatively. The paired median within-patient change was 10 points (95 % CI for the median difference, 10–25), and this improvement was statistically significant (p = 0.005). Consistent with these summary statistics, KPS improved in 10 of 12 patients (83.3 %) and was unchanged in 2 (16.7 %); no patients experienced worsening in this regard.

**Table 3:** Comparison of KPS scores before and after surgery.

		Assessment				
	Group	Preoperative	Postoperative	Median Difference	95 % CI^a^	p-value^b^
	**Unilateral Hemilaminectomy (N=12)**			10	10; 25	**0.005**
	Mean (±SD)	73.3 (±20.2)	87.5 (±14.8)			
	Median [Q1-Q3]	80 [60–90]	90 [85–100]			
	Min – Max	40–100	50–100			
	**Total Laminectomy (N=36)**			5	0; 20	**<0.001**
	Mean (±SD)	62.8 (±23.7)	72.2 (±26.0)			
	Median [Q1-Q3]	60 [40–80]	80 [55–100]			
	Min - Max	10–100	10–100			

a 95 % Confidence interval from a nonparametric bootstrap with 10,000 resamples.

b Paired Wilcoxon signed rank test with continuity correction.

In the TL group (N = 36), the mean KPS increased from 62.8 (±23.7) preoperatively to 72.2 (±26.0) postoperatively; the median increased from 60 [40–80] to 80 [55–100], with a preoperative and postoperative range of 10–100. The paired median change was 5 points (95 % CI for the median difference, 0–20), which was also statistically significant (p < 0.001). Categorically, KPS improved in 18 of 36 patients (50.0 %), remained unchanged in 16 (44.4 %), and worsened in 2 (5.6 %) patients.

In Table 4, Table 5 are shown preoperative difference of KPS and McCormick grade for intradural intramedullary and intradural extramedullary tumors.

**Table 4:** Comparison of KPS scores before and after surgery, per tumor localisation.

		Assessment				
	Tumor localisation	Preoperative	Postoperative	Median Difference	95 % CI^a^	p-value^b^
	**Intradural intramedullary (N=10)**			10	0; 20	0.14
	Mean (±SD)	53.0 (±24.1)	62.0 (±26.6)			
	Median [Q1-Q3]	50 [40–60]	60 [50–80]			
	Min – Max	10–90	10–100			
	**Intradural extramedullary**^c^**(N=31)**			0	0; 20	**<0.001**
	Mean (±SD)	70.6 (±22.2)	82.9 (±21.6)			
	Median [Q1-Q3]	70 [60–90]	90 [70–100]			
	Min – Max	10–100	10–100			

a 95 % Confidence interval from a nonparametric bootstrap with 10,000 resamples.

b Paired Wilcoxon signed rank test with continuity correction.

c Includes intradural extramedullary tumors (n = 28) and intradural extramedullary tumors with extradural expansion (n = 3).

**Table 5:** Comparison of neurological outcomes (McCormick grade) between timepoints, per tumor localisation.

		Assessment		
Group	McCormick	Preoperative^a^	Postoperative^a^	p-value^b^
**Intradural intramedullary (N=10)**				0.003
	1	0 (0.0 %)	1 (10.0 %)	
	2	2 (20.0 %)	3 (30.0 %)	
	3	3 (30.0 %)	4 (40.0 %)	
	4	4 (40.0 %)	1 (10.0 %)	
	5	1 (10.0 %)	1 (10.0 %)	
**Intradural extramedullary**^c^**(N=31)**				0.26
	1	5 (16.1 %)	13 (41.9 %)	
	2	12 (38.7 %)	12 (38.7 %)	
	3	7 (22.6 %)	2 (6.5 %)	
	4	5 (16.1 %)	2 (6.5 %)	
	5	2 (6.5 %)	2 (6.5 %)	

a n (%).

b Stuart-Maxwell Marginal Homogeneity Test.

c Includes intradural extramedullary tumors (n = 28) and intradural extramedullary tumors with extradural expansion (n = 3).

**Fig. 5:**
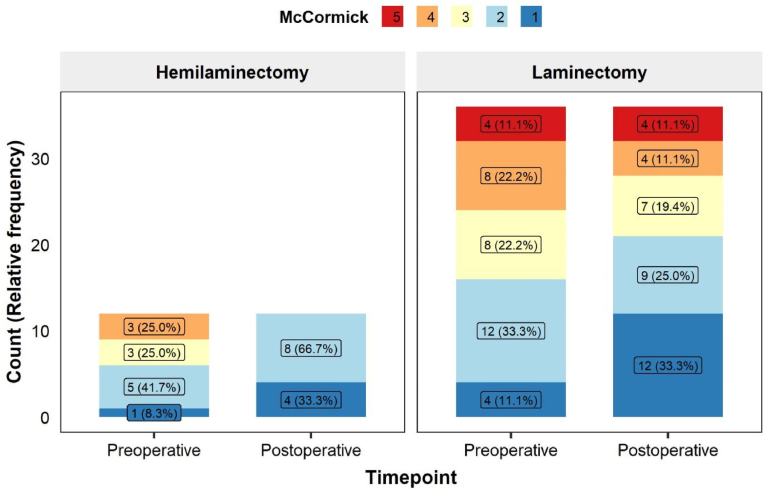
Stacked bar plot for McCormick grade score between groups across timepoints.

McCormick grade between timepoints is shown in Fig. 5. Significant neurological recovery was observed in both groups as assessed by the Stuart-Maxwell Marginal Homogeneity Test. For UHL, the improvement was statistically significant (p = 0.029). Postoperatively, 100 % of patients achieved the best two functional categories (Grade 1 or 2), a considerable gain from the 50.0 % who were in these categories preoperatively. The TL group also showed a significant positive shift in McCormick grade distribution (p = 0.004). The proportion of patients categorized as Grade 1 (best outcome) more than doubled, increasing from 11.1 % preoperatively to 33.3 % postoperatively.

## 4 Discussion

Our cohort analysis demonstrates that **unilateral hemilaminectomy is non-inferior to total laminectomy** across several outcomes. Specifically, we found no statistically significant difference between the two surgical approaches regarding safety and effectiveness for **intradural or extradural spinal tumor resection**, functional neurological status (as measured by McCormick and KPS scores), or overall postoperative complication rates.

These findings are comparable to the results of recent systematic reviews and meta-analyses. In the meta-analysis by Piñeiro et al., 2025, which included 12 studies and 1047 patients, no difference was found in the sense of complete resection rates between UHL and TL. They showed that UHL was associated with a significantly reduced risk of postoperative neurological deterioration and complications, as well as shorter operative time, less intraoperative blood loss, and reduced length of hospital stay (Liao et al., 2023). Similarly, the meta-analysis by Lei et al., 2021), covering 13 studies with 1424 patients, showed that UHL leads to shorter operations, reduced blood loss, faster mobilization, and a lower risk of postoperative spinal deformity compared to laminectomy.

Our results are also similar to those of Chen et al. (2024). published in Frontiers in Neurology, who compared UHL with laminoplasty in 100 patients undergoing resection of spinal schwannomas. They found that functional outcomes (McCormick and KPS scores) were comparable between the two techniques. In our study in both groups preoperative and postoperative McCormick and KPS scores have statistically significant improvement. Both the UHL and TL groups demonstrated significant postoperative functional improvement; however, there was no statistically significant difference in the magnitude of improvement between the two cohorts. In our study, operative duration and length of hospitalization did not differ significantly between groups. Conversely, Chen et al., 2024 reported significantly shorter operative time and shorter postoperative hospitalization in the UHL group, also complete resection rates were comparable between the two techniques (Naganawa et al., 2011). Their findings confirm that UHL might bring perioperative advantages without compromising oncological or functional results, but we did not observe the same results in our study.

A large series was published by Liao et al., 2023), where 916 tumors were removed with UHL. They achieved a complete resection rate of 97.8 %, with a significantly shorter surgery time, less blood loss, and faster recovery for patients.

A study published in 2024 showed in thoracic extramedullary tumors that minimally invasive UHL provides equally successful neurological outcomes as TL, like our study, but with shorter hospital stay and less blood loss (Okubo et al., 2024). We have shown that UHL can be also used for intramedullary tumors, especially it can be challenging for larger and surgically complex tumors, like in case of a intramedullary hemangioblastoma shown in Fig. 2. Previously, Onishi et al., 2025 also emphasized that UHL is a safe option for tumors located close to the spinal cord, with the use of adequate microneurosurgical techniques (Onishi et al., 2025).

Based on available literature, UHL can be considered an equal alternative to TL, particularly when maintaining the posterior ligamental complex of the spine and bony structures, which can contribute to maintaining spinal alignment. Its advantages along with reduced surgical stress fit into the general trend of modern neurosurgery towards minimally invasive techniques. However, the choice of approach must be individualized, because larger, medially located or highly vascularized tumors may require wider exposure through laminectomy or laminoplasty (Naganawa et al., 2011 and Goodarzi et al., 2020).

Moreover, recent literature has shown that postoperative changes in the muscles damaged during surgery and their correlation with surgical outcomes. However, there is need for more definitive evidence to substantiate these findings (Fu et al., 2020) (Seçen et al., 2023). Crucially, the inherent value of UHL lies in its capacity for **tissue preservation**, which is the primary driver for its adoption in modern neurosurgery. By preserving the posterior tension band and minimizing damage to the bilateral paraspinal musculature (the main cause of iatrogenic spinal deformity and chronic axial pain following total laminectomy), UHL fundamentally addresses the long-term morbidity associated with traditional open surgery. It is clear that UHL causes less muscle damage, but the clinical significance of this finding requires further evidence. In a study, the posterior unilateral approach for resection of cervical dumbbell-shaped schwannoma effectively preserved the cervical spinal angle, leading to favorable clinical outcomes and satisfactory sagittal alignment. This technique maintained postoperative cervical range of motion, and maintained acceptable sagittal alignment with satisfactory functional and clinical outcomes at the final follow-up (Okubo et al., 2024). (Yasuoka et al., 1982).

For tumors located laterally, with clear separation from the spinal cord, UHL is a safe option. But for tumors in the midline, large tumors crossing both sides, or those with a major vascular component, standard TL or combined approaches like laminoplasty may still can be required to avoid iatrogenic spinal cord injury. Several studies confirm that the limited surgical corridor of UHL can be a limiting factor in such complex cases (Chen et al., 2024; Seçen et al., 2023). (Naganawa et al., 2011; Yeo et al., 2011).

### 4.1 Limitations

Our study has several limitations. The retrospective design introduces a risk of selection bias, since the choice of surgical approach depended on the surgeon's preference and tumor characteristics. The number of patients is small so differences between groups might not be detected. The tumors were very different in size, type, and location, which makes it harder to make a strong conclusion for each subgroup. The follow-up period was also short to evaluate long-term outcomes, with statistically significant between groups, which can influence the McCormick score, KPS due the factor of time. Spinal stability and postoperative deformity were not addressed in this study. Future studies should include larger, multicenter prospective studies with standardized outcome measures. Endoscopy may further improve more safety and promote the use of minimally invasive techniques.

## 5 Conclusion

Our study supports the current literature by demonstrating that UHL might have same results as TL in removing the same size tumors with similar functional outcomes and complication rates. However, the approach must remain individualized based on tumor complexity, radiological findings and tumor type.

## Declaration of competing interest

The authors declare that they have no known competing financial interests or personal relationships that could have appeared to influence the work reported in this paper.
